# Apolipoprotein B48 as a Marker of Residual Coronary Risk: Diagnostic Insights from a Comparative Analysis with Ankle-Brachial Index

**DOI:** 10.24546/0100497875

**Published:** 2025-10-21

**Authors:** KENTA MORI, ASUKA MONOBE, SADATSUGU OKUMA, TATSURO ISHIDA

**Affiliations:** 1 Department of General Internal Medicine, Toyooka Hospital, Hyogo, Japan; 2 Fujirebio Inc., Tokyo, Japan; 3 Division of Cardiovascular Medicine, Kobe University Graduate School of Medicine, Kobe, Japan; 4 Division of Nursing Practice, Kobe University Graduate School of Health Sciences, Kobe, Japan

**Keywords:** Apolipoprotein B48, Ankle-brachial index, Coronary artery disease, Diabetes mellitus

## Abstract

Apolipoprotein B48 (ApoB48) may be an indicator of residual cardiovascular risk beyond conventional lipid measures. However, its performance for detecting coronary artery disease (CAD), alone and in combination with the ankle-brachial index (ABI), remains to be investigated. This cross-sectional study (358 patients; 299 and 59 with and without CAD, respectively) assessed the value of ApoB48 (cutoff: ≥4.5 μg/mL) and ABI (cutoff: <0.9) in detecting CAD. Subgroup analyses were performed for patients with diabetes mellitus, hypertension, dyslipidemia, and low levels of low-density lipoprotein-cholesterol (LDL-C) (<100 mg/dL). Baseline characteristics, including lipid profiles and biomarker levels, were compared between patients with and without CAD. Patients with CAD exhibited significantly higher ApoB48 levels compared to those without (5.1 ± 3.2 vs. 4.0 ± 2.2 μg/mL, respectively, p = 0.001); there were no significant differences in ABI values. The sensitivity and specificity of ABI alone for CAD were 16.7% and 81.4%, respectively, while those for ApoB48 alone were 48.2% and 61.0%, respectively. Combining both markers improved sensitivity to 55.5%, though specificity declined to 47.5%. Subgroup analyses revealed that ApoB48 maintained superior sensitivity across groups with diabetes, hypertension, dyslipidemia, and low levels of LDL-C. Lipid parameters (LDL-C, non-high density lipoprotein-cholesterol, and triglycerides) showed minimal discriminatory power between patients with and without CAD. ApoB48 demonstrates superior sensitivity for CAD detection compared to ABI, particularly in high-risk patients. While combining ApoB48 and ABI enhances sensitivity, it compromises specificity, suggesting the need for balanced diagnostic strategies. ApoB48 may be a valuable marker of residual cardiovascular risk, particularly in patients with well-controlled LDL-C or comorbid metabolic conditions.

## INTRODUCTION

Coronary artery disease (CAD) remains a leading cause of morbidity and mortality worldwide, despite advances in preventive strategies and lipid-lowering therapies. The residual cardiovascular risk observed even in patients receiving intensive statin treatment highlights the need for novel biomarkers that reflect atherogenic processes beyond traditional lipid parameters (1, 2).

ApoB48 is a structural component of chylomicrons and their remnants, which contribute to atherosclerotic plaque formation through postprandial and fasting lipid metabolism disturbances (3, 4). Direct measurement of chylomicrons and their remnants is technically challenging in routine clinical practice due to their rapid clearance and heterogeneous composition. Therefore, ApoB48—being a unique structural component synthesized exclusively in the intestine—has been introduced as a clinically applicable surrogate marker for evaluating remnant lipoprotein metabolism and its atherogenic potential (5–7).

The ankle-brachial index (ABI) is a simple, non-invasive diagnostic tool widely used for assessing peripheral artery disease. However, its diagnostic performance for CAD detection is limited, especially in asymptomatic populations and those with metabolic abnormalities Combining ABI with additional biomarkers may enhance diagnostic sensitivity for CAD; nonetheless, the optimal approach remains to be determined (8, 9).

The aim of this study was to evaluate the diagnostic performance of ApoB48 and ABI, both individually and in combination, for CAD detection in a diverse patient cohort. Furthermore, we assessed their performance across subgroups of patients with diabetes mellitus, hypertension, dyslipidemia, and low LDL-C levels, reflecting populations at high residual cardiovascular risk. By elucidating the relative contributions of ApoB48 and ABI, this study seeks to inform strategies for more accurate risk stratification and management of CAD.

## MATERIALS AND METHODS

### Patients

Patients with and without CAD admitted to Kobe University Hospital (Kobe, Japan) from April 2008 to March 2012 were eligible. Coronary lesions were defined by ≥75% narrowing of the coronary luminal diameter, measured by coronary angiography. Patients were registered only once during the study period. Exclusion criteria were emergency admission, heart failure (New York Heart Association functional class 4), cancer in the past 5 years, pulmonary hypertension, kidney failure (serum creatinine concentration: >2.0 mg/dL or hemodialysis), and active inflammation (serum C-reactive protein concentration: >1 mg/dL).

Patients with systolic blood pressure >140 mm Hg or diastolic blood pressure >90 mm Hg were diagnosed with hypertension. Blood pressure was also recorded for patients treated with antihypertensive drugs. Patients with fasting serum glucose >126 mg/dl or hemoglobin A1c value >6.5% (National Glycohemoglobin Standardization Program) were diagnosed with diabetes mellitus, according to the clinical guidelines of the Japan Diabetes Society. Diabetes was also recorded for patients treated with antidiabetic drugs. Patients with high serum LDL-C concentration (≥140 mg/dL) were diagnosed with dyslipidemia, according to the Japan Atherosclerosis Society Guidelines for Prevention of Atherosclerotic Cardiovascular Diseases (10).

Dyslipidemia was also recorded for patients treated with antihyperlipidemic drugs or was defined according to the Japan Atherosclerosis Society guidelines (11). With regard to smoking status, patients were categorized as never smoked, former smoker, or current smoker. Former smokers had not smoked for ≥1 years. Among the enrolled subjects, those with missing values in ApoB48 and lipid profiles were excluded.

### Ethical considerations

The study was conducted in accordance with the ethical principles stipulated in the Declaration of Helsinki and the Ethical Guidelines for Clinical Research, enforced by the Ministry of Health, Labour and Welfare of Japan from July 31, 2008. The study protocol was approved by the institutional review board of Kobe University Graduate School of Medicine, Japan. All patients provided written informed consent prior to enrolment.

### Biochemical analyses

Serum samples were collected after overnight fasting at the point of initial admission. Serum samples were stored at −80°C until use, and biochemical analyses were performed using standard techniques (12). ApoAI, ApoB, and ApoE levels were measured using the immunoturbidity method (Sekisui Medical, Tokyo, Japan). Remnant-like particle cholesterol concentration was measured by immunoadsorption using the Jimro-II assay kit (Otsuka Pharmaceutical, Tokyo, Japan). ApoB48 levels were measured using the chemiluminescent enzyme immunoassay with anti-human ApoB48 monoclonal antibodies (Fujirebio, Tokyo, Japan) (12).

### Statistical analysis

Data are expressed as mean ± standard deviation or frequencies (%). Chi-squared test was used to compare categorical data between groups. All statistical analyses were performed using Stata 13.1 (Stata Corp., College Station, TX, USA), and MS Excel (Microsoft Corporation, Redmond, WA, USA). A p-value < 0.05 indicates a statistically significant difference. Analyses were performed using complete-case data. Cases with missing data for relevant variables were excluded from the corresponding analyses. Given the large sample size, the impact of missing data was minimal and did not affect the results.

## RESULTS

An ABI value < 0.9 denoted positivity (13, 14). Based on data from our previous study, the cutoff value for positive ApoB48 levels was set at 4.5 mg/dL (4, 15). The diagnostic performance for detecting CAD was evaluated using the sensitivity and specificity of ABI and ApoB48.

### Overall patient population

In the overall patient cohort (n = 358), patients with CAD (n = 299) were more likely to be male and had higher waist circumference, body mass index (BMI), and triglyceride (TG) levels, and lower HDL-C levels compared to those without CAD (n = 59). ApoB48 levels were significantly higher in patients with CAD compared to those without (5.1 ± 3.2 μg/mL vs. 4.0 ± 2.2 μg/mL, respectively, p = 0.001). There was no significant difference observed in LDL-C (95.2 ± 27.5 vs. 101.7 ± 27.2 mg/dL, respectively, p = 0.101) or non-HDL cholesterol (117.3 ± 31.6 vs. 120.3 ± 30.9 mg/dL, respectively, p = 0.508). In addition, ABI values did not differ significantly (1.1 ± 0.2 vs. 1.1 ± 0.2, respectively, p = 0.539) (Table I).

**Figure 1:**
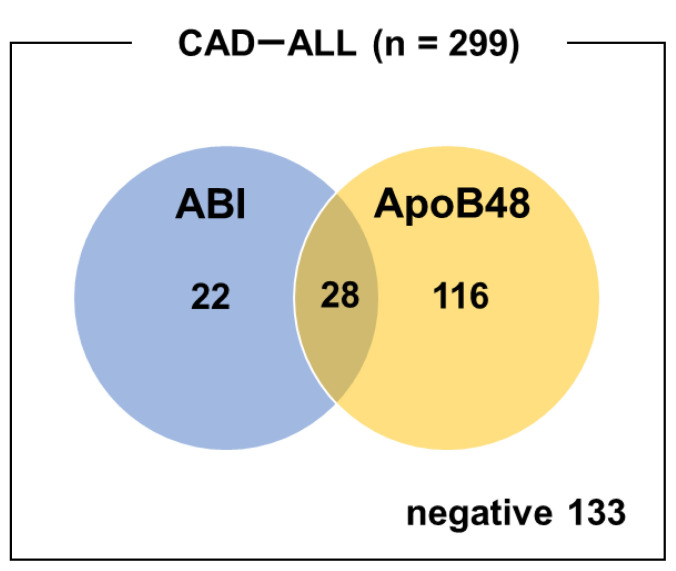
Venn diagram of ABI-positive and ApoB48-positive cases in patients with CAD. The blue and orange circles represent cases with a positive ABI (<0.9) and those with elevated ApoB48 levels (≥4.5 μg/mL), respectively. The intersection indicates patients who were positive for both criteria.

For the overall population, the sensitivity and specificity values of ABI (<0.9) alone for CAD were 16.7% and 81.4%, respectively. ApoB48 (≥4.5 μg/mL) alone demonstrated higher sensitivity (48.2%), but lower specificity (61.0%). Combining both markers improved sensitivity to 55.5%, whereas specificity was decreased to 47.5% (Table II, Figure 1).

### Diabetes subgroup

In the diabetes subgroup (n = 190), patients with CAD were older and had higher BMI compared to those without CAD. ApoB48 levels were significantly higher in patients with CAD (5.2 ± 3.2 μg/mL vs. 3.4 ± 1.3 μg/mL, respectively, p < 0.001), whereas HDL-C levels were lower (45.4 ± 11.7 vs. 50.6 ± 9.9 mg/dL, respectively, p = 0.035). There were no significant differences in LDL-C, non-HDL cholesterol, Tcho, or ApoB levels between patients with and without CAD. ABI values did not differ significantly (1.0 ± 0.2 vs. 1.0 ± 0.2, respectively, p = 0.586) (Table III).

**Figure 2:**
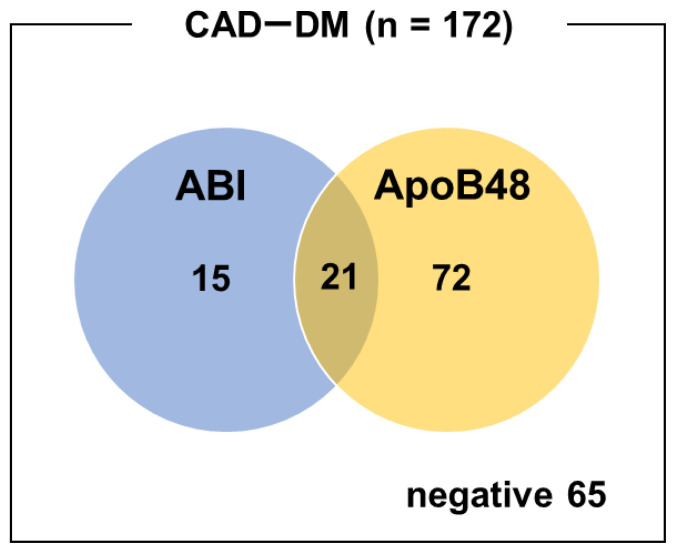
Venn diagram of ABI-positive and ApoB48-positive cases in patients with both CAD and diabetes. The blue and orange circles represent cases with a positive ABI (<0.9) and those with elevated ApoB48 levels (≥4.5 μg/mL), respectively. The intersection indicates patients who were positive for both criteria.

In this subgroup, ABI alone had a sensitivity and specificity of 20.9% and 66.7% for detecting CAD, respectively, while the corresponding values of ApoB48 were 53.5% and 77.8%, respectively. Combining both markers improved sensitivity to 62.2%, whereas specificity was decreased to 55.6% (Table IV, Figure 2).

### Hypertension subgroup

In the hypertension subgroup (n = 295), patients with CAD were more likely to receive statin therapy and had significantly higher ApoB48 levels compared to those without CAD (5.0 ± 3.1 μg/mL vs. 3.9 ± 2.2 μg/mL, respectively, p = 0.006). There were no significant differences observed in LDL-C, HDL-C, TG, or non-HDL cholesterol levels between patients with and without CAD. Moreover, ABI values did not differ significantly (1.1 ± 0.2 vs. 1. 1 ± 0.2, respectively, p = 0.866) (Table V).

**Figure 3:**
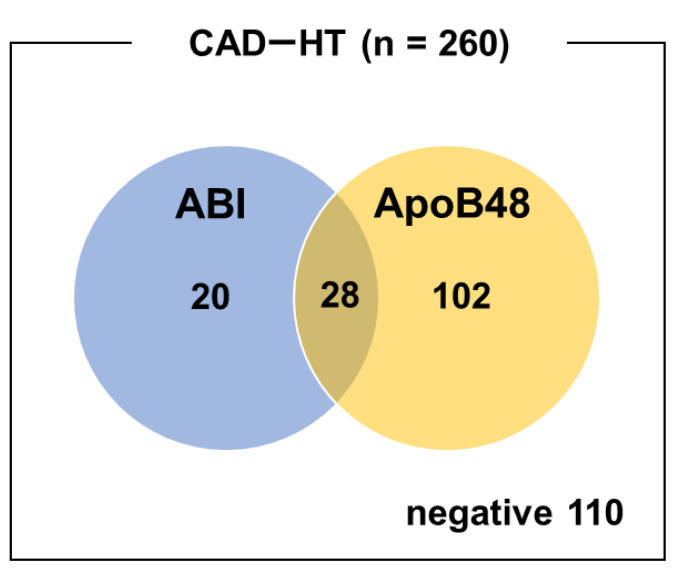
Venn diagram of ABI-positive and ApoB48-positive cases in patients with both CAD and hypertension. The blue and orange circles represent cases with a positive ABI (<0.9) and those with elevated ApoB48 (≥4.5 μg/mL). The intersection indicates patients positive for both criteria.

ABI alone showed a sensitivity of 18.5% and specificity of 74.3% for CAD, while ApoB48 alone had a sensitivity of 50.0% and specificity of 65.7%. Combining both markers improved sensitivity to 57.5%, whereas specificity was decreased to 48.6% (Table VI, Figure 3).

### Dyslipidemia subgroup

In the dyslipidemia subgroup (n = 276), patients with CAD had significantly higher ApoB48 levels than those without CAD (5.0 ± 3.1 μg/mL vs. 3.9 ± 2.2 μg/mL, respectively, p = 0.006) and were more frequently prescribed statins. There were no significant differences in LDL-C, HDL-C, TG, or non-HDL cholesterol levels. Furthermore, ABI values did not differ significantly (1.1 ± 0.2 vs. 1.1 ± 0.2, respectively, p = 0.866) (Table VII).

**Figure 4:**
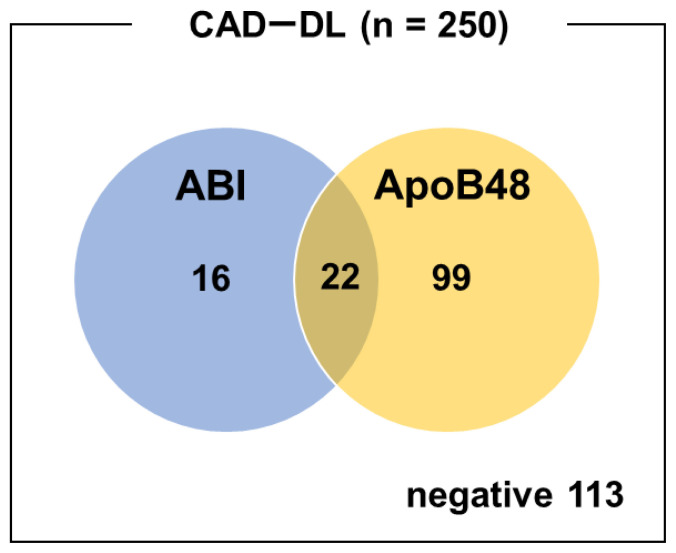
Venn diagram of ABI-positive and ApoB48-positive cases in patients with both CAD and dyslipidemia. The blue and orange circles represent cases with a positive ABI (<0.9) and those with elevated ApoB48 levels (≥4.5 μg/mL), respectively. The intersection indicates patients who were positive for both criteria.

In this subgroup, ABI alone demonstrated sensitivity and specificity of 15.2% and 73.1% for CAD, respectively, while ApoB48 alone showed sensitivity of 48.4% and specificity of 61.5%. Combining both markers resulted in sensitivity and specificity of 54.8% and 46.2%, respectively (Table VIII, Figure 4).

### Low LDL-C (<100 mg/dL) subgroup

In the low LDL-C subgroup (n = 202), patients with CAD were older, had higher BMI, larger waist circumference, and lower HDL-C levels compared to those without CAD. ApoB48 levels were significantly higher in patients with CAD (4.7 ± 2.9 μg/mL vs. 3.3 ± 1.9 μg/mL, respectively, p < 0.001), while LDL-C, non-HDL cholesterol, and Tcho levels did not differ significantly. In addition, ABI values were similar between the groups (1.1 ± 0.2 vs. 1.1 ± 0.2, respectively, p = 0.761) (Table IX).

**Figure 5:**
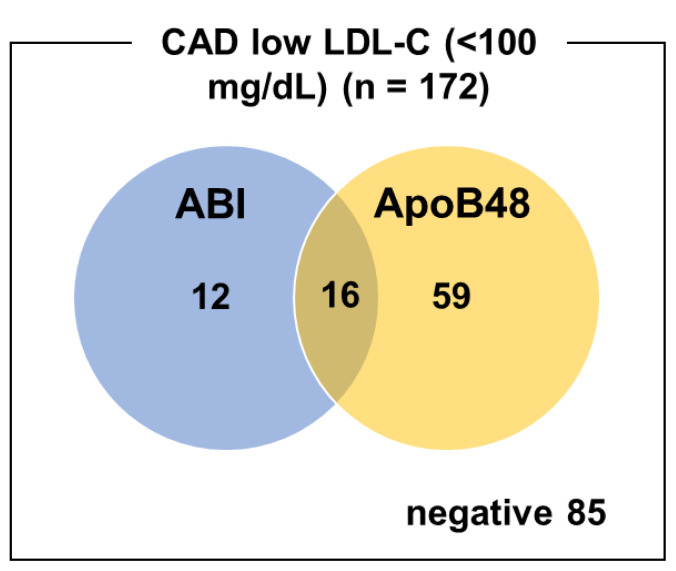
Venn diagram of ABI-positive and ApoB48-positive cases in patients with both CAD and low LDL-C levels (<100 mg/dL). The blue and orange circles represent cases with a positive ABI (<0.9) and those with elevated ApoB48 levels (≥4.5 μg/mL), respectively. The intersection indicates patients who were positive for both criteria.

ABI alone had a sensitivity of 16.3% and specificity of 76.7% for CAD, while ApoB48 alone had a sensitivity of 43.6% and specificity of 76.7%. Combining both markers improved sensitivity to 50.6%, whereas specificity was decreased to 60.0% (Table X, Figure 5).

## DISCUSSION

This study evaluated the diagnostic performance of ApoB48 and ABI, both individually and in combination, for the detection of CAD in a cohort of 358 patients, including various subgroups—such as those with diabetes, hypertension, dyslipidemia, and low LDL-C. The results demonstrated that ApoB48 consistently exhibited higher sensitivity than ABI across the entire cohort and within subgroups stratified by diabetes mellitus, hypertension, dyslipidemia, and low LDL-C levels. Furthermore, while the combination of ApoB48 and ABI improved sensitivity, it invariably reduced specificity.

The low sensitivity of ABI in this study is consistent with prior findings; this evidence highlights its limited utility for detecting CAD, especially in asymptomatic populations or those with metabolic disturbances (13, 14). In contrast, ApoB48, a marker reflecting intestinal chylomicron remnants, demonstrated superior diagnostic performance. This aligns with previous reports indicating that elevated ApoB48 levels represent increased postprandial and fasting chylomicron remnant particles, which play a significant role in atherogenesis (3, 4).

Importantly, ApoB48 levels were significantly higher in patients with CAD compared to those without CAD across all subgroups, whereas LDL-C, non-HDL cholesterol, and other lipid parameters showed minimal differences between these patient groups. This finding reinforces the hypothesis that ApoB48 serves as a marker of residual cardiovascular risk, particularly in patients with well-controlled LDL-C levels or those receiving intensive lipid-lowering therapy. Prior studies conducted by Mori et al. (5, 6) demonstrated that ApoB48 is associated with both incident and progressive CAD, even in patients undergoing statin therapy. Elevated ApoB48 levels have also been linked to the progression of new atherosclerotic lesions after percutaneous coronary intervention, suggesting its utility as a predictor of residual risk not captured by traditional lipid metrics (5, 6).

While combining ApoB48 with ABI increased sensitivity for detecting CAD, it reduced specificity. This observation underscores the necessity of balancing sensitivity and specificity when designing diagnostic strategies, especially in populations with low disease prevalence or when considering screening programs (16, 17). Diagnosis of CAD in chronic- and asymptomatic patients is often difficult in clinical settings, because it sometimes requires interventional procedures or radiological imaging with contrast agents. Our findings indicate that combination of ApoB48 with ABI may provide a non-invasive strategy for detecting CAD, which results in an overall decrease in patients undergoing invasive screening tests.

The subgroup analyses provided further insights. In patients with diabetes, hypertension, dyslipidemia, and low levels of LDL-C—characterized by complex metabolic profiles and elevated cardiovascular risk—ApoB48 demonstrated robust diagnostic performance. The persistently higher ApoB48 levels recorded in patients with CAD, despite minimal differences in LDL-C levels and other lipid indices, highlight its potential clinical value in identifying residual risk that conventional markers may overlook (18–20).

In addition to ABI, several non-invasive vascular screening tools, including pulse wave velocity (PWV) and carotid ultrasonography, have been recommended by international and national guidelines for cardiovascular risk assessment—particularly in patients with metabolic disorders or subclinical atherosclerosis. PWV is a validated measure of arterial stiffness and is recognized as an independent predictor of cardiovascular events by the European Society of Cardiology (ESC) and the Japan Atherosclerosis Society (JAS) guidelines (16, 21). Likewise, carotid intima-media thickness (IMT) and plaque evaluation are widely endorsed as surrogate markers of systemic atherosclerosis and future coronary events (22). Comparative assessment of ApoB48 with these established modalities may clarify its relative diagnostic value and position within multimodal risk stratification strategies.

**Figure 6:**
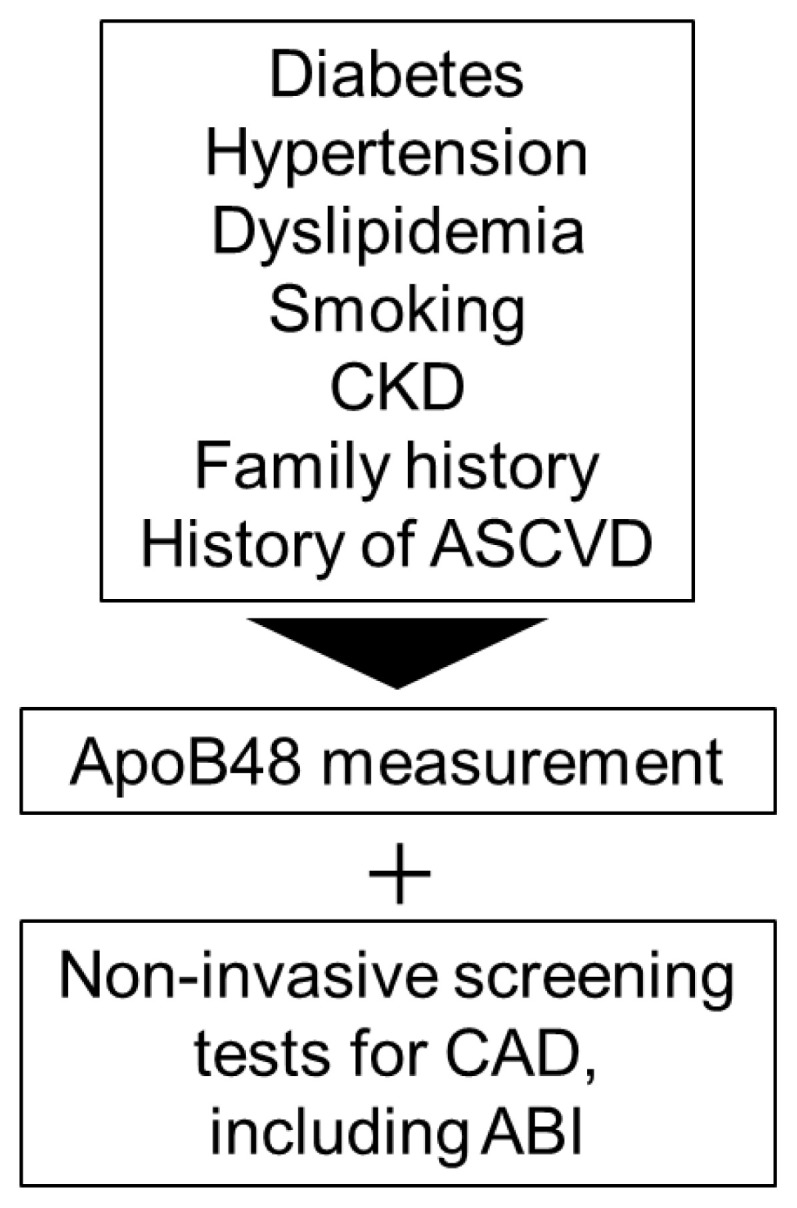
Positioning of ApoB48 measurement in the screening of CAD. In patients with high-risk factors for CAD, measurement of ApoB48 levels may be considered in conjunction with non-invasive screening tests, such as ABI.

Our findings suggest that ApoB48 is a superior risk marker for CAD, particularly in high-risk patient populations. Its measurement may be particularly valuable in identifying residual cardiovascular risk in patients with well-controlled LDL-C levels or metabolic comorbidities. The combination of ApoB48 and ABI enhances sensitivity, while reducing specificity; thus, it is necessary to consider an optimal diagnostic algorithm that integrates clinical background for accurate assessment. Based on the findings of this study, it is desirable to develop new screening guidelines that recommend the combined use of ABI and ApoB-48, particularly in patients with diabetes mellitus, to enhance early detection and risk assessment (Figure 6) (23). Further prospective studies are warranted to validate these findings and clarify the role of ApoB48 in individualized risk assessment and management of CAD.

## Figures and Tables

**Table I:** Characteristics of patients with or without CAD

	Patients without CAD n = 59	Patients with CAD n = 299	p-value
**Male, n (%)**	38 (64.4)	245.0 (81.9)	0.002
**Age (years)**	66.4 ± 9.1	68.5 ± 9.8	0.117
**Weight (kg)**	65.8 ± 12.0	65.8 ± 12.0	<0.001
**Waist (cm)**	83.2 ± 10.4	89.7 ± 8.9	<0.001
**BMI (kg/m** **^2^** **)**	22.5 ± 3.6	24.9 ± 3.6	<0.001
**HT, n (%)**	35 (59.3)	260 (87.0)	<0.001
**DM, n (%)**	18 (30.5)	172 (57.5)	<0.001
**DL, n (%)**	26 (44.1)	250 (83.6)	<0.001
**Current smoking, n (%)**	12 (20.3)	61 (20.4)	0.991
**LDL-C (mg/dL)**	101.7 ± 27.2	95.2 ± 27.5	0.101
**HDL-C (mg/dL)**	53.1 ± 11.9	47.4 ± 13.2	0.002
**Non-HDL-C (mg/dL)**	120.3 ± 30.9	117.3 ± 31.6	0.508
**Tcho (mg/dL)**	173.4 ± 31.9	164.8 ± 30.9	0.068
**TG (mg/dL)**	115.5 ± 56.0	137.5 ± 69.9	0.010
**ApoAI (mg/dL)**	133.1 ± 20.0	127.7 ± 22.2	0.068
**ApoAII (mg/dL)**	31.3 ± 6.3	31.3 ± 6.1	0.964
**ApoB (mg/dL)**	73.9 ± 17.3	72.9 ± 17.3	0.682
**ApoB48 (μg/mL)**	4.0 ± 2.2	5.1 ± 3.2	0.001
**RLP-C (mg/dL)**	7.0 ± 4.1	8.1 ± 5.5	0.098
**ABI**	1.1 ± 0.2	1.1 ± 0.2	0.539
**Statin, n (%)**	16 (27.1)	205 (68.6)	<0.001
**DPP4, n (%)**	4 (6.8)	24 (8.0)	0.744
**EPA, n (%)**	1 (1.7)	23 (7.7)	0.092
**Fibrate, n (%)**	1 (1.7)	10 (3.3)	0.502
**Ezetimibe, n (%)**	0	11 (3.7)	0.135
**Insulin, n (%)**	1 (1.7)	27 (9.0)	0.055

DL, dyslipidemia; DM, diabetes mellitus; DPP4, dipeptidyl peptidase 4; EPA, eicosapentaenoic acid; HDL-C, high-density lipoprotein-cholesterol; HT, hypertension; RLP-C, remnant-like particle-cholesterol.

The p-values are based on χ^2^ test (categorical values) or Student’s *t*-test (continuous variables). Data represent the mean ± standard deviation, unless otherwise specified.

**Table II:** Diagnostic performance of ABI and ApoB48 for the detection of CAD

	Patients with CAD	Patients without CAD	Row total
ABI-positive	50	11	61
ABI-negative	249	48	297
Colum total	299	59	358
	SN = 16.7% (50/299)	SP = 81.4% (48/59)	

ApoB48-positive	144	23	167
ApoB48-negative	155	36	191
Colum total	299	59	358
	SN = 48.2% (144/299)	SP = 61.0% (36/59)	

ABI-posivive or ApoB48-positive	166	31	197
ABI-negative and ApoB48-negative	133	28	161
Colum total	299	59	358
	SN = 55.5% (166/299)	SP = 47.5% (28/59)	

SN, sensitivity; SP, specificity.

This table summarizes the diagnostic performance of ABI (<0.9) and ApoB48 (≥4.5 μg/mL) for detecting CAD (patients with CAD: n = 358; patients without CAD: n = 59). “ABI-positive or ApoB48-positive” represents cases that were positive for either marker; while “ABI-negative and ApoB48-negative” includes cases that were negative for both.

**Table III:** Characteristics of patients with or without CAD and with diabetes

	Patients without CAD n = 18	Patients with CAD n = 172	p-value
**Male, n (%)**	17 (94.4)	155.0 (90.1)	0.168
**Age (years)**	64.2 ± 9.9	66.9 ± 10.0	0.247
**Weight (kg)**	62.7 ± 12.5	67.0 ± 12.5	0.168
**Waist (cm)**	86.6 ± 10.5	90.6 ± 9.0	0.113
**BMI (kg/m** **^2^** **)**	23.5 ± 3.8	25.3 ± 3.7	0.046
**HT, n (%)**	13 (72.2)	170 (98.8)	<0.001
**DM, n (%)**	–	–	–
**DL, n (%)**	10 (55.6)	167 (97.1)	<0.001
**Current smoking, n (%)**	6 (33.3)	40 (23.3)	0.501
**LDL-C (mg/dL)**	89.4 ± 25.1	94.4 ± 26.5	0.395
**HDL-C (mg/dL)**	50.6 ± 9.9	45.4 ± 11.7	0.035
**Non-HDL-C (mg/dL)**	108.2 ± 29.4	117.2 ± 31.3	0.199
**Tcho (mg/dL)**	158.8 ± 31.5	162.7 ± 30.6	0.596
**TG (mg/dL)**	115.4 ± 66.1	142.0 ± 73.6	0.098
**ApoAI (mg/dL)**	130.8 ± 22.1	124.6 ± 21.0	0.229
**ApoAII (mg/dL)**	31.4 ± 6.1	31.1 ± 5.9	0.827
**ApoB (mg/dL)**	68.2 ± 16.5	72.9 ± 17.3	0.236
**ApoB48 (μg/mL)**	3.4 ± 1.3	5.2 ± 3.2	<0.001
**RLP-C (mg/dL)**	6.5 ± 4.6	8.3 ± 5.7	0.110
**ABI**	1.0 ± 0.2	1.0 ± 0.2	0.586
**Statin, n (%)**	6 (33.3)	136 (79.1)	<0.001
**DPP4, n (%)**	4 (22.2)	26 (15.1)	0.627
**EPA, n (%)**	0	12 (7.0)	0.118
**Fibrate, n (%)**	0	6 (3.5)	0.272
**Ezetimibe, n (%)**	0	8 (4.7)	0.204
**Insulin, n (%)**	1 (5.6)	26 (15.1)	0.063

**Table IV:** Diagnostic performance of ABI and ApoB48 for the detection of CAD in patients with diabetes

	Patients with CAD	Patients without CAD	Row total
ABI-positive	36	6	42
ABI-negative	136	12	148
Colum total	172	18	190
	SN = 20.9% (36/172)	SP = 66.7% (12/18)	

ApoB48-positive	92	4	96
ApoB48-negative	80	14	94
Colum total	172	18	190
	SN = 53.5% (92/172)	SP = 77.8% (14/18)	

ABI-posivive or ApoB48-positive	107	8	115
ABI-negative and ApoB48-negative	65	10	75
Colum total	172	18	190
	SN = 62.2% (107/172)	SP = 55.6% (10/18)	

This table summarizes the diagnostic performance of ABI (<0.9) and ApoB48 (≥4.5 μg/mL) for detecting CAD (patients with CAD: n = 190; patients without CAD: n = 18). “ABI-positive or ApoB48-positive” represents cases that were positive for either marker; while “ABI-negative and ApoB48-negative” includes cases that were negative for both.

**Table V:** Characteristics of patients with or without CAD and with hypertension

	Patients without CAD n = 35	Patients with CAD n = 260	p-value
**Male, n (%)**	24 (68.6)	213 (81.9)	0.062
**Age (years)**	66.5 ± 9.6	68.6 ± 9.9	0.234
**Weight (kg)**	60.1 (12.1)	65.0 ± 12.1	0.018
**Waist (cm)**	86.1 (10.4)	89.5 ± 9.2	0.056
**BMI (kg/m** **^2^** **)**	23.6 (3.7)	24.8 ± 3.7	0.040
**HT, n (%)**	–	–	–
**DM, n (%)**	13 (37.1)	170 (65.4)	0.001
**DL, n (%)**	20 (57.1)	238 (91.5)	0.000
**Current smoking, n (%)**	8 (22.9)	54 (20.8)	0.776
**LDL-C (mg/dL)**	98.1 ± 28.9	95.8 ± 27.8	0.622
**HDL-C (mg/dL)**	50.9 ± 9.5	47.8 ± 13.1	0.062
**Non-HDL-C (mg/dL)**	117.5 ± 32.5	117.8 ± 32.0	0.953
**Tcho (mg/dL)**	168.4 ± 33.9	165.8 ± 31.4	0.636
**TG (mg/dL)**	119.7 ± 58.1	136.3 ± 69.3	0.090
**ApoAI (mg/dL)**	131.8 ± 19.4	128.3 ± 22.4	0.269
**ApoAII (mg/dL)**	31.5 ± 6.6	31.3 ± 6.2	0.877
**ApoB (mg/dL)**	73.1 ± 17.2	73.1 ± 17.4	0.995
**ApoB48 (μg/mL)**	3.9 ± 2.2	5.0 ± 3.1	0.006
**RLP-C (mg/dL)**	7.0 ± 4.3	8.0 ± 5.5	0.157
**ABI**	1.1 ± 0.2	1.1 ± 0.2	0.866
**Statin, n (%)**	14 (40)	192 (73.8)	<0.001
**DPP4, n (%)**	2 (5.7)	26 (10.0)	0.417
**EPA, n (%)**	1 (2.9)	21 (8.1)	0.270
**Fibrate, n (%)**	0	10 (3.8)	0.238
**Ezetimibe, n (%)**	0	9 (3.5)	0.264
**Insulin, n (%)**	0	26 (10.0)	0.050

**Table VI:** Diagnostic performance of ABI and ApoB48 for the detection of CAD in patients with hypertension

	Patients with CAD	Patients without CAD	Row total
ABI-positive	48	9	57
ABI-negative	212	26	238
Colum total	260	35	295
	SN = 18.5% (48/260)	SP = 74.3% (26/35)	

ApoB48-positive	130	12	142
ApoB48-negative	130	23	153
Colum total	260	35	295
	SN = 50.0% (130/260)	SP = 65.7% (23/35)	

ABI-posivive or ApoB48-positive	150	18	168
ABI-negative and ApoB48-negative	110	17	127
Colum total	260	35	295
	SN = 57.5% (150/260)	SP = 48.6% (17/35)	

This table summarizes the diagnostic performance of ABI (<0.9) and ApoB48 (≥4.5 μg/mL) for detecting CAD (patients with CAD: n = 260; patients without CAD: n = 35). “ABI-positive or ApoB48-positive” represents cases that were positive for either marker; while “ABI-negative and ApoB48-negative” includes cases that were negative for both.

**Table VII:** Characteristics of patients with or without CAD and with dyslipidemia

	Patients without CAD n = 26	Patients with CAD n = 260	p-value
**Male, n (%)**	16 (61.5)	213 (81.9)	0.062
**Age (years)**	65.5 ± 10.3	68.6 ± 9.9	0.234
**Weight (kg)**	61.4 ± 12.1	65.0 ± 12.1	0.018
**Waist (cm)**	86.4 ± 11.1	89.5 ± 9.2	0.056
**BMI (kg/m** **^2^** **)**	23.9 ± 3.8	24.8 ± 3.7	0.040
**HT, n (%)**	20 (76.9)	260 (100)	<0.001
**DM, n (%)**	10 (38.5)	170 (65.4)	0.001
**DL, n (%)**	–	–	–
**Current smoking, n (%)**	6 (23.1)	54 (20.8)	0.776
**LDL-C (mg/dL)**	98.5 ± 32.1	95.8 ± 27.8	0.622
**HDL-C (mg/dL)**	50.3 ± 10.7	47.8 ± 13.1	0.062
**Non-HDL-C (mg/dL)**	120.2 ± 36.4	117.8 ± 32.0	0.953
**Tcho (mg/dL)**	170.6 ± 37.2	165.8 ± 31.4	0.636
**TG (mg/dL)**	135.7 ± 64.4	136.3 ± 69.3	0.090
**ApoAI (mg/dL)**	133.7 ± 20.1	128.3 ± 22.4	0.269
**ApoAII (mg/dL)**	33.4 ± 5.9	31.3± 6.2	0.877
**ApoB (mg/dL)**	74.6 ± 20.0	73.1 ± 17.4	0.995
**ApoB48 (μg/mL)**	4.0 ± 2.0	5.0 ± 3.1	0.006
**RLP-C (mg/dL)**	7.9 ± 4.8	8.0 ± 5.5	0.157
**ABI**	1.0 ± 0.2	1.1 ± 0.2	0.866
**Statin, n (%)**	16 (61.5)	192 (73.8)	<0.001
**DPP4, n (%)**	1 (3.8)	26 (10.0)	0.417
**EPA, n (%)**	1 (3.8)	21 (8.1)	0.270
**Fibrate, n (%)**	1 (3.8)	10 (3.8)	0.238
**Ezetimibe, n (%)**	0	9 (3.5)	0.264
**Insulin, n (%)**	0	26 (10.0)	0.050

**Table VIII:** Diagnostic performance of ABI and ApoB48 for the detection of CAD in patients with dyslipidemia

	Patients with CAD	Patients without CAD	Row total
ABI-positive	38	7	45
ABI-negative	212	19	231
Colum total	250	26	276
	SN = 15.2% (38/250)	SP = 73.1% (19/26)	

ApoB48-positive	121	10	131
ApoB48-negative	129	16	145
Colum total	250	26	276
	SN = 48.4% (121/250)	SP = 61.5% (16/26)	

ABI-posivive or ApoB48-positive	137	14	151
ABI-negative and ApoB48-negative	113	12	125
Colum total	250	26	276
	SN = 54.8% (137/250)	SP = 46.2% (12/26)	

This table summarizes the diagnostic performance of ABI (<0.9) and ApoB48 levels (≥4.5 μg/mL) for detecting CAD (patients with CAD: n = 250; patients without CAD: n = 26). “ABI-positive or ApoB48-positive” represents cases that were positive for either marker; while “ABI-negative and ApoB48-negative” includes cases that were negative for both.

**Table IX:** Characteristics of patients with or without CAD and with low LDL-C levels (<100 mg/dL)

	Patients without CAD n = 30	Patients with CAD n = 172	p-value
**Male, n (%)**	22 (73.3)	141 (82.0)	0.268
**Age (years)**	65.3 ± 9.3	69.6 ± 9.7	0.025
**Weight (kg)**	56.6 ± 11.7	65.1 ± 11.7	<0.001
**Waist (cm)**	81.9 ± 8.6	89.3 ± 9.2	<0.001
**BMI (kg/m** **^2^** **)**	22.0 ± 3.4	24.6 ± 3.4	<0.001
**HT, n (%)**	17 (56.7)	147 (85.5)	<0.001
**DM, n (%)**	13 (43.3)	104 (60.5)	0.079
**DL, n (%)**	14 (46.7)	155 (90.1)	<0.001
**Current smoking, n (%)**	7 (23.3)	31 (18.0)	0.492
**LDL-C (mg/dL)**	80.1 ± 16.4	75.9 ± 14.4	0.198
**HDL-C (mg/dL)**	53.7 ± 12.0	48.6 ± 14.1	0.048
**Non-HDL-C (mg/dL)**	96.3 ± 19.3	95.8 ± 16.0	0.892
**Tcho (mg/dL)**	150.0 ± 22.6	144.6 ± 18.3	0.233
**TG (mg/dL)**	104.9 ± 62.2	123.0 ± 59.8	0.155
**ApoAI (mg/dL)**	131.4 ± 21.2	128.6 ± 23.3	0.522
**ApoAII (mg/dL)**	31.1 ± 6.7	30.4 ± 5.8	0.595
**ApoB (mg/dL)**	60.7 ± 11.4	61.5 ± 9.5	0.738
**ApoB48 (μg/mL)**	3.3 ± 1.9	4.7 ± 2.9	<0.001
**RLP-C (mg/dL)**	5.8 ± 4.0	6.3 ± 4.0	0.573
**ABI**	1.1 ± 0.2	1.1 ± 0.2	0.761
**Statin, n (%)**	9 (30.0)	14 (8.1)	<0.001
**DPP4, n (%)**	3 (10.0)	14 (8.1)	0.735
**EPA, n (%)**	0	14 (8.1)	0.105
**Fibrate, n (%)**	1 (3.3)	4 (2.3)	0.743
**Ezetimibe, n (%)**	0	7 (4.1)	0.261
**Insulin, n (%)**	1 (3.3)	17 (9.9)	0.245

**Table X:** Diagnostic performance of ABI and ApoB48 for the detection of CAD in patients with low LDL-C levels (<100 mg/dL)

	Patients with CAD	Patients without CAD	Row total
ABI-positive	28	7	35
ABI-negative	144	23	167
Colum total	172	30	202
	SN = 16.3% (28/172)	SP = 76.7% (23/30)	

ApoB48-positive	75	7	82
ApoB48-negative	97	23	120
Colum total	172	30	202
	SN = 43.6% (75/172)	SP = 76.7% (23/30)	

ABI-posivive or ApoB48-positive	87	12	99
ABI-negative and ApoB48-negative	85	18	103
Colum total	172	30	202
	SN = 50.6% (87/172)	SP = 60.0% (18/30)	

This table summarizes the diagnostic performance of ABI (≤0.9) and ApoB48 levels (≥4.5 μg/mL) for detecting CAD (patients with CAD: n = 172; patients without CAD: n = 30). “ABI-positive or ApoB48-positive” represents cases that were positive for either marker; while “ABI-negative and ApoB48-negative” includes cases that were negative for both.
